# Initial Surgery in Tailoring Treatment for Children With Stage II and III Wilms’ Tumor: An Experience From Resource Challenged Settings

**DOI:** 10.14740/wjon876w

**Published:** 2015-10-26

**Authors:** Ossama M. Zakaria, Emad N. Hokkam, Karam Al Sayem, Mohamed Yasser I. Daoud, Hazem M. Zakaria, Fouad Sedky, Seba H. Graiz, Saleh A. Moussa, Hamed A. Al Wadaani

**Affiliations:** a Department of Surgery, College of Medicine, King Faisal University, Al Ahsa, KSA; b Department of Surgery, Faculty of Medicine, Suez Canal University, Egypt; c Department of Surgery, College of Medicine, Dammam University, KSA; d Prince Saud bin Jalawi Hospital, MOH, Al Ahsa, KSA

**Keywords:** Wilms’ tumor, Children, Chemotherapy and surgery, Cost-effectiveness, Resource challenged settings

## Abstract

**Background:**

Although Wilms’ tumor (WT) is ranked first among primary childhood’s renal neoplasm, controversy still exists regarding the best approach for its management. The study aimed at evaluating the role of initial surgery in treatment of stage II and III pediatric WT as a part of the short administration schedule as in National Wilms’ Tumor Study (NWTS)-4 and evaluating its effectiveness compared to the long administration schedule.

**Methods:**

The study included 30 children who were primarily diagnosed as stage II and III WT. They were divided into two equal groups. Group I (n = 15) included those children who had undergone neoadjuvant chemotherapy followed by surgery and postoperative chemotherapy, while group II (n = 15) included those children who had undergone primary surgery as an initial management followed by chemotherapy. After a mean postoperative follow-up period of 14 ± 5 months, clinical and radiological evaluation was performed to all patients.

**Results:**

In group I, 10 patients were preoperatively diagnosed as stage II and five patients as stage III while in group II, 11 patients were proved to be stage II and four patients were stage III. After a follow-up period, clinical and radiological evaluation using CT was performed to all patients. In patients with stage II, evidence of recurrence was noted in three patients of group I whereas no patient showed any evidence of recurrence in group II. In patients with stage III, rebound increase in size was seen in two patients in group I and only one patient in group II.

**Conclusions:**

Initial surgical intervention with appropriate adjuvant therapy has better outcomes than the neoadjuvant chemotherapy and delayed surgery for children primarily diagnosed as stage II and III WT. Moreover, it may act as a short administration schedule for the treatment as it is not less effective than the long administration schedule and can be administered at a substantially lower total treatment cost.

## Introduction

Renal neoplasms in childhood are usually malignant, the most common being Wilms’ tumor (WT) [1]. The incidence varies from 10.9 per million in the USA to 2.5 per million in Chinese [2]. Therapeutic approach varies geographically as it sometimes depends largely on the cost-effectiveness of the chosen policy [3].

In Europe and some extra-European countries, patients are treated according to Societe Internationale d’Oncologie Pediatrique/International Society of Paediatric Oncology (SIOP) protocol which advocates preoperative chemotherapy for 4 - 6 weeks relying on initial diagnostic imaging, followed by surgery [4, 5].

Most of the United States and Canada follow the National Wilms’ Tumor Study Group (NWTSG) protocol which mandates primary nephrectomy for all cases with the exception of the large unilateral or bilateral tumors, while further adjuvant therapy is given based on surgical and pathologic findings [6-8]. The fundamental differences existing between these two large cooperative multinational trials are primary surgery in NWTSG versus initial or neoadjuvant chemotherapy in SIOP [5].

Despite the debate over whether chemotherapy should be given before surgery [9-11], the clinical outcomes are excellent in both groups, and productive debate continues on the merits of each approach [4].

The issue here is which approach should supersede the other as a treatment option specifically in stage II and III. Some researchers tried to find out the answer [12]. At present, the decision to follow either approach is subjective in centers that are not a part of these groups.

The aim of the study is to evaluate the impact of initial surgery (NWTSG) as a guide to determine an accurate stage and to tailor treatment for children who were primarily diagnosed as stage II and III WT as a short administration treatment schedule compared with the long administration schedule in SIOP and its effect on lowing the total treatment cost in resource challenged setting nations.

## Patients and Methods

The study recruited 30 children who were primarily diagnosed as stage II and III WT over a period of 8 years (2004 - 2012). The initial assessment included clinical examination as well as laboratory investigations including complete blood count, urine analysis specially urine catecholamines to rule out neuroblastoma, serum urea and creatinine levels. Abdominal ultrasonography and CT were performed to all patients to confirm the diagnosis and to exclude other abdominal masses not originating in the kidney. Chest CT and radiographs were used for detection of lung metastases.

Inclusion criteria were CT proved unilateral WT (stage II and III) children. While excluded were those children presenting with other abdominal malignancies and/or other renal lesions such as hydronephrosis or cystic disease. Patients with hematogenous metastasis were also excluded.

Children were divided into two equal groups. In group I (n = 15), preoperative chemotherapy was decided according to CT diagnosis, whereas precise staging was assigned after surgery. In group II (n = 15), surgical intervention was done as an initial management followed by chemotherapy. Commonly employed chemotherapeutic agents included dactinomycin, vincristine, doxorubicin, cyclophosphamide, etoposide, and carboplatin. Chemotherapy dosage depends on the particular stage of the disease and the child (Table 1, 2) [13, 14]. In group I, surgical exploration was performed as soon as the child health was optimized, usually within 6 weeks after the initial diagnosis.

**Table 1:** Chemotherapeutic Agents Used for WT Treatment

Drug name	Pediatric dose
Dactinomycin	0.015 mg/kg IV push qd for 5 days
Vincristine	1.5 mg/m^2^ IV q1-3 weeks, not to exceed 2 mg/dose
Cyclophosphomide	1.2 - 2.2 g/m^2^ IV qd for 1 - 3 days
Etoposide	100 mg/m^2^ IV qd for 5 days
Doxorubicin (adriamycin)	45 mg/m^2^ IV

**Table 2:** Chemotherapeutic Regimens in Relation to the Stage of WT

Stage	Chemotherapeutic regimen
Stage II (FH), stage III (FH)	DD-4A (AMD, VCR, and DOX; 24 weeks
Stage II or stage III (focal or diffuse anaplasia)	I (VCR + CPM + E; 24 weeks)

FH: favorable histology; AMD: dactinomycin; VCR: vincristine; DOX: doxorubicin; CPM: cyclophosphamide: E: etoposide.

Surgery entailed radical excision of the tumor whenever amenable. A transverse abdominal incision was done to provide adequate exposure, from the tip of the 12th rib on the involved side to the lateral rectus border on the opposite side. Exploration of the contralateral kidney with biopsy as needed was carried out first; reflection of colon and complete mobilization of kidney are required for adequate visualization and manual inspection of front and back surfaces of the kidney. Radical nephrectomy was done whenever possible. Metal clips were left to identify residual masses in stage III patients.

Histopathological examination was performed for all surgically removed specimens. CT follow-up and clinical evaluation was performed to all patients to detect recurrence in stage II and follow-up of the size of the residual mass in stage III. For statistical analysis, SPSS 20 was used. Collected data were tabulated and analyzed statistically using χ^2^ analysis. Continuous variable was analyzed using the independent sample *t*-test. P values less than 0.5 were considered statistically significant.

## Results

Thirty patients were studied; they were 17 males and 13 females with the ratio of 1.3:1. The age of the studied patients in group I ranged from 1.1 years to 16 years (mean = 6.8 ± 1.3), while in group II, it ranged from 1.3 years to 14 years (mean = 7.3 ± 1.6) with no statistical significant difference between the two groups. Palpable abdominal mass was the first presentation in 19 patients (63.3%), recurrent abdominal pain in five patients (16.7%) and hypertension in six patients (20%).

In group I, 10 patients were preoperatively diagnosed as stage II, while the remaining five patients were preoperatively diagnosed as stage III. After a mean postoperative follow-up period of 14 ± 5 months (mean ± SD), three patients with preoperatively diagnosed stage II showed CT evidenced recurrence. On the other hand, remission was noted in three patients with stage III whereas rebound increase in size was seen in the remaining two.

In group II, 11 patients were proved to be stage II, whereas four patients were stage III. The postoperative follow-up was identical to that of group I. Yet, no patients with stage II showed any evidence of recurrence. Nevertheless, only one patient with stage III showed relapse. In this group, chemotherapy regimens were modulated taking in consideration the histopathological grade found at biopsy.

Table 3 shows the relapses in both groups.

**Table 3:** Comparative Relapse Rate Between Groups I and II

Relapse	Group I	Group II	P value
Stage II	3 (20%)	0	< 0.02
Stage III	2 (13.3%)	1	< 0.04

**Figure 1:**
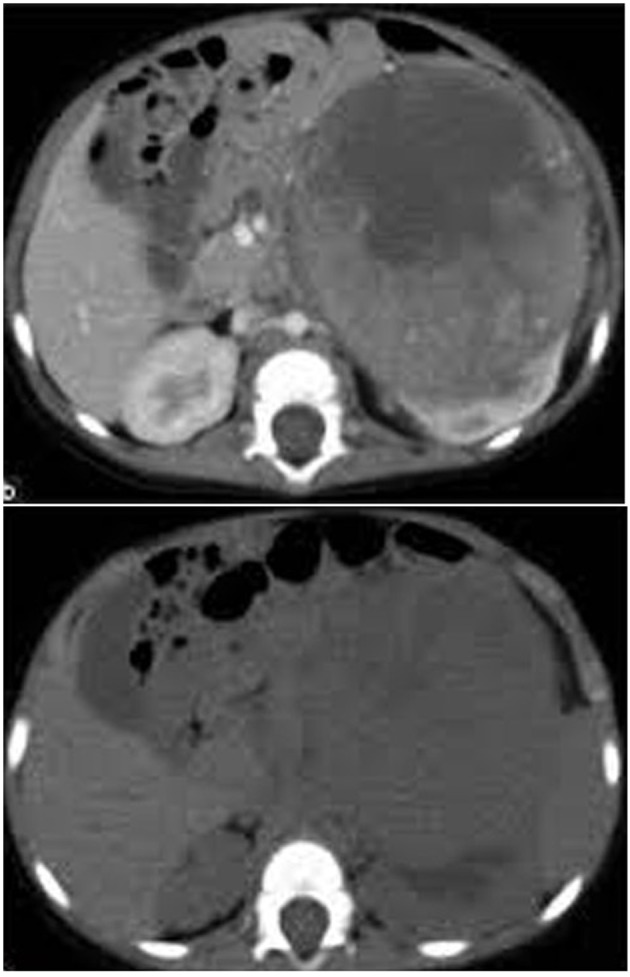
CT image showing a left renal mass with enhancing renal parenchyma posteriorly. Lobulated tumor thrombus is present in the left renal vein and IVC.

In group I, five patients (33.3%) were found to be understaged at histopathological examination with CT accuracy of 66.7% (P < 0.03) compared to surgical exploration and biopsy. This was due to unresectable tumor margins in spite of being stage II on CT (Fig. 1). On the other hand, histopathological examination confirmed free margins in all patients with stage II in group II (P < 0.01).

The overall histopathological results revealed favorable histology (tubular predominance) in 26 patients (86.7%) whereas unfavorable histology (anaplasia, rhabdoid and clear cell sarcoma) in four patients (13.3%). The most commonly encountered complications among our patients after chemotherapy were tabulated (Table 4).

**Table 4:** Complications of Chemotherapy Among the Studied Patients

Complication	Number of patients
Bone marrow depression	9 (30%)
Bowel obstruction	4 (13.3%)
Hepatic dysfunction	6 (20%)
Interstitial pneumonitis	2 (6.7%)
Cardiomyopathy	1 (3.3%)

## Discussion

Management of WT is evolving at a rapid pace and remains a paradigm for multimodal cancer therapy [5, 15]. The two main study groups are SIOP and NWTSG. SIOP has advocated preoperative radiotherapy in the first two trials and then used chemotherapy in the following four trials for 4 - 6 weeks. The advantage of SIOP protocol is to reduce the incidence of tumor rupture, intra-peritoneal tumor spillage, obtain a more favorable stage distribution, and in turn, reduce the treatment burden. Beside that this protocol gives the opportunity to judge responsiveness of the tumor to the standard regimen of chemotherapy so that risk stratification and treatment adjustments are feasible in postoperative period. However, the potential disadvantages of SIOP are not obtaining untreated tissue for proper histopathological study, treatment of a benign condition with chemotherapy and treatment of a different malignant disease with the wrong chemotherapy as well as the relative long time of administration schedule that may be more expensive [9, 12, 13].

The NWTSG approach recommends up-front surgery with certain exceptions: bilateral tumors, tumors in a solitary or horseshoe kidney, extension of tumor thrombus in the supra-hepatic cava or heart, and extensive metastatic disease causing respiratory distress. There are mainly two advantages of this approach: accurate and early complete staging and obtaining an untreated tumor specimen that can be subjected for tissue diagnosis and other biological prognostic studies as well as the short administration schedule that may decrease the cost of treatment [3]. The disadvantage of this approach is higher rate of surgical complications like tumor rupture and intra-operative spillage [12-15].

In this study, we have applied the two protocols for children who were primary staged as stage II and III according to the CT findings. Neoadjuvant chemotherapy was adopted as the first line of management in group I children which coincided with researchers who primarily stemmed their experience from the application of SIOP protocols including a period of preoperative chemotherapy followed by surgery and a period of postoperative chemotherapy [14, 16, 17]. In group II, we adopted the National Wilms’ Tumor Society protocol (NWTS) with surgery as the first line of treatment followed by chemotherapeutic application [18, 19]. This group has the advantage of histological confirmation of the disease as well as accurate staging during surgery. During the operation, the contralateral kidney was also explored to ensure that the disease was indeed unilateral and lymph node dissection was carried out [20]. We did not perform transcutaneous biopsy for any of our cases with the concept that it may complicate the treatment in accordance with the same concept in a previous study [21, 22].

The study results showed that patients in group I were having a significantly less success rate as compared to those in group II. Such results were contradictory to previous published results of SIOP protocols [21] while coincided with those of NWTSG [23, 24].

Tumor histology and stage are the two most significant prognostic factors for patients with WT [25]. In group I, the preoperative chemotherapy alters the tumor’s histological features [26], thus making the pathologist’s job to assign the subtype of histopathology and stage very difficult while in patients with group II, the pathologist could properly identify and stage the tumor.

Generally, children can tolerate the acute toxicities of chemotherapeutic drugs better than adults [27]. However, they are more susceptible for delayed side effects of chemotherapy like growth problems, infertility and neuropsychological dysfunction [28].

In our study, the most commonly encountered complication is bone marrow depression (30%) followed by bowel obstruction (13.3%). This is in agreement with other recent studies [29, 30].

In conclusion, initial surgical resection remains a crucial part in treatment of stage II & III WT patients as a short administration schedule that will reduce the cost-effectiveness of treatment especially in resource challenged settings as in our case. It can provide a local primary tumor control, accurate staging, proper histological interpretation and possibly controlling the metastatic spread. However, patient selection for surgery is an important determinant for successful outcome.
